# Parental burnout among Somali mothers: Associations with mental health, perceived social support, and sociodemographic factors

**DOI:** 10.1371/journal.pgph.0002501

**Published:** 2023-10-19

**Authors:** Juweria N. L. Abshir, Fatumo Osman, Gallad Dahir, Anton Dahlberg

**Affiliations:** 1 School of Health and Welfare, Dalarna University, Falun, Sweden; 2 School of Public Health and Research, Somali National University, Mogadishu, Somalia; 3 Child Health and Parenting (CHAP), Department of Public Health and Caring Sciences, Uppsala University, Uppsala, Sweden; PLOS: Public Library of Science, UNITED STATES

## Abstract

Parenthood can be defined by the contradiction that it is one of the most satisfying yet stressful experiences in life. Many parents experience stress during parenthood, and some to the extent that they display symptoms of parental burnout. Nevertheless, research on parental burnout is scant and many studies have only examined the condition in Western settings. The aim of this study was to examine parental burnout among Somali mothers in Mogadishu, Somalia, and its association with certain psychological, psychosocial, and sociodemographic factors. In this cross-sectional study, questionnaire data were collected through the measurements Parental Burnout Assessment and Patient Health Questionnaire 9, as well as through social and demographic questions. A total of 882 Somali mothers in Mogadishu participated. The analysis methods used were univariate, bivariate, and multiple linear regression analysis. The results revealed that the mean parental burnout score was low in the sample. Additionally, a significant association was found between higher levels of parental burnout and higher levels of depression, perceived lack of social support, being unmarried, having a low monthly household income, and when the youngest child was of school-age.

## Introduction

Parental burnout is a context-specific condition that has its origin in research on job burnout. However, due to its specific nature, the condition has more distinct consequences in the particular sphere of parenting [1]. When defining parental burnout, it is important to separate it from *parenting stress*. Parenting stress is common and even necessary in one’s parenting role. However, when the parenting stress persistently and substantially overwhelms the parent’s resources to cope, that is when parental burnout occurs [2]. In this respect, parental burnout is, according to Mikolajczak, Gross, and Roskam [3], defined as a state in which the parent experiences intense exhaustion and chronic stress related to their parental role, where the experienced demands surpass the available resources. Parental burnout manifests through four main symptoms, typically displayed through four stages: emotional exhaustion, emotional distancing from one’s child(ren), loss of pleasure in the parental role, and contrast with previous parental self. The risk of developing parental burnout has been enhanced over recent years [4]. However, research on parental burnout is still in its infancy, and there is a general lack of research [2].

Parental burnout has shown to impair the parent’s ability to perform their parental duties, which has a negative impact on the parent-child relationship. For instance, it can lead parents to exhibit violent- and neglectful behaviour towards their children [2, 4]. Interestingly, parental burnout has shown to greatly affect mothers [5, 6]. Research has, moreover, shown that parental burnout is frequently accompanied by other mental disorders, such as depression and anxiety [7–9], and in some cases, the condition might cause mental disorders [5]. There are also indications that parental burnout is associated with lower perceptions of social support [8] and partner parental support [10]. In addition, the correlation between parental burnout and different sociodemographic factors is yet to be fully understood [11]. Recent studies on parental burnout have identified some sociodemographic factors (e.g., financial situation and the age of the youngest child) which may either act as risk- or protective factors [11–13]. Other associated factors are being a woman, being a young mother, being a single parent, or having many children [14]. Other research indicates that sociodemographic factors are less predictive of parental burnout [15–17]. However, recent research concludes that being a mother is a risk factor for parental burnout, and mothers are more vulnerable to this condition in contrast to fathers [5, 6, 18, 19]. Moreover, parental burnout has mainly been examined among parents in high-income and Western settings, while parents in other contexts, especially in Sub-Saharan settings, have been neglected in the discourse and research on parental burnout [19]. This has resulted in low scientific understanding of the condition in a non-Western setting. Additionally, the geographical aspect should be considered when examining parental burnout, as it can affect the score and which factors are associated with the condition. Parenting in African countries usually occurs in a collectivistic culture, which is characterised by a high level of interdependence where interconnectedness is essential. This means that the parental responsibility does not merely fall on the parents [19]. Instead, older siblings, relatives, and neighbours have a responsibility towards the child, which usually is to have an educational role by transmitting values, good morals, and skills to the child. In this regard, the parental role is shared with others and not only confined to the parent in collectivistic cultures. However, despite the differences in parenting arrangements in certain African countries, when compared with other countries, very little research has been conducted from this context on parental burnout. This becomes concerning, since despite the role-sharing arrangement, the difficult socio-economic conditions and social problems in many African countries can still make it difficult and stressful for parents to fulfil their parental duties [19].

Thus, in this study, the attention will be turned towards mothers in Mogadishu, Somalia, since little is known about parental burnout among mothers in this context. From a scientific perspective, Mogadishu becomes an interesting setting to examine parental burnout, since the city struggles with many social problems, such as maternal mortality and indiscriminate attacks, which leaves families vulnerable [20, 21]. Therefore, with this chosen setting, this study can help fill the existing research gap on parental burnout.

### Aim

This study aims to examine parental burnout among Somali mothers in Mogadishu, Somalia, and its association with certain psychological, psychosocial, and sociodemographic factors.

## Methods

### Participants

The data collection took place between August and September 2021, as part of a larger research project, in which the present study was one part. Participants were recruited from nine government-owned Maternity and Child Health centres (MCHs) in Mogadishu, Somalia with the help of four experienced Somali speaking data collectors and one of the co-authors (GD). The data collectors’ tasks were to make the questionnaire available to the participants and help them with reading the questions and marking their responses due to low literacy levels. Two of the co-authors (FO, GD) were supervising the data collectors, through daily contact, providing feedback and helping when challenges arose. Lastly, the supervisor accompanied the data collectors to ensure that the data was collected ethically.

In total, data were collected between August and September 2021 from 900 Somali women who visited any of the nine MHCs. As only mothers were of interest in this study, eighteen responses from soon-to-be mothers were excluded, resulting in a sample of 882 mothers. Information on the study was given written and verbally to mothers who were waiting to receive care or received care. Only the mothers who gave written consent—or verbal when not able to read or write—participated in the study. To prevent individual participants from the risk of being identified in the dataset, all collected data were anonymous. Ethical approval for this study was obtained by the Somali National University, School of Public Health (SNU/SPHR/005/2021) and permission was also sought at district and health facility level.

### Inclusivity in global research

Additional information regarding the ethical, cultural, and scientific considerations specific to inclusivity in global research is included in the Supporting Information (S1 Checklist).

### Instruments

The data used in this study were collected using a digital self-reported questionnaire in Somali. Questionnaires were answered digitally through designated mobile phones, using the data collection program KoboToolbox. Before the data collection was conducted, the questionnaire was pilot tested on eight Somali mothers in Mogadishu. During the pilot testing, it was observed that the mothers occasionally found the questions in the Parental Burnout Assessment (PBA) measurement to be strange and would ask the data collector how a mother could feel negatively towards her children.

#### Parental Burnout Assessment, PBA

The PBA consists of 23 questions that are divided into four symptom dimensions of parental burnout. The first dimension is *Emotional exhaustion*, which consists of nine questions such as “I feel completely run down by my role as a parent”. The next dimension is *Contrast with the previous parental self*, which consists of six questions such as “I am ashamed of the parent that I have become”. The third dimension is *Loss of pleasure in the parental role*, which consists of five questions such as “I do not enjoy being with my child(ren)”. The fourth dimension is *Emotional distancing from one’s child(ren)*, which consists of three questions such as “I am no longer able to show my child(ren) how much I love them”. The questionnaire was answered using a seven-point frequency scale from zero to six (0 = never, 6 = daily). Since the participants in this study consisted of Somali mothers, the questions were translated to Somali by following the fourth steps according to World Health Organizations’ process of translation [22]. The PBA was first translated into Somali by the second author and checked for errors by the third author. Subsequently, a research member fluent in both English and Somali performed the back translation. The back translation was then discussed with the reference group, who agreed to pilot test the measure on the target population. During the pilot testing, it was observed that some mothers occasionally found the questions in the PBA measurement to be unusual and would inquire of the data collector how a mother could feel negatively towards her children.

Internal consistency was assessed for the study sample, providing high alpha-values overall (Full scale ⍺ = .95; Exhaustion ⍺ = .89; Contrast in parental self ⍺ = .89; Feelings of being fed up ⍺ = .91; Emotional distancing ⍺ = .32). The Emotional distancing subscale alone showed low internal consistency. An inspection of the subscale revealed specifically low correlations for the item "I do what I’m supposed to do for my child(ren), but nothing more”. With this item removed, the subscale ⍺ increased to .73. For this study, only the Full scale (PBA total score) with all items included was used.

#### Patient Health Questionnaire, PHQ-9

To assess the level of depression among the participants in this study the PHQ-9 was used, as it has shown to be applicable in different contexts around the globe [23, 24]. The PHQ-9 is a short self-assessment questionnaire used for screening for major depressive disorder and assessing the current level of depressive symptoms [23]. The PHQ-9 has been translated into different languages and has shown great validity when tested. In 2016, a Somali version of the PHQ-9 was assessed psychometrically by Nallusamy and colleagues [24], and showed to have strong internal consistency, homogeneity, and reliability.

#### Perceived social support and support from husband

In this study, social support was assessed based on the mothers’ perceptions of being cared for by others and having a trusted network to rely on. Additionally, the study examined the mothers’ views on support from their husbands. The questions included access to help and support from family, relatives, friends, or others when needed, partner parental support, specifically the perceived support received from the mothers’ husbands.

### Data analysis

All analyses were carried out using SPSS and R. Multicollinearity among independent variables was assessed using Pearson’s correlations and Spearman’s rank correlations. Correlation coefficients above .70 were excluded from further analyses, to avoid multi-collinearity within the regression model. Age and number of children had a correlation >.70 and subsequently age was excluded from the regression analyses. This, since the variable number of children was proven to be of higher scientific interest based on previous research [12, 13, 19]. No other variables reached the threshold and were thus kept in the regression analysis. Associations between PBA scores and independent variables were analysed through multiple linear regression.

## Results

For a breakdown of sociodemographic variables in the sample, please see Table 1. The majority of the Somali mothers in this study were married (95.4%), which resulted in a small variety in marital status. Interestingly, when examining the monthly household income, the variable was mostly divided into two categories where the majority of the mothers either had a monthly income of less than 100 USD (41.8%) or around 100 USD (48.8%). Furthermore, the mean age of the mothers was around 28 years. Lastly, the mean level in the PHQ-9 was 4, which indicated a low level of depression among the mothers. Nonetheless, differences in individual responses have been distinguished, which showed that the depression scores ranged from 0 to 21. This suggested that there were differences in the degree of depression among the individual participants in this study, while the overall level of depression remained low.

**Table 1:** Sociodemographic variables (n = 882).

Variables	n	%	Min-Max
Marital status			
Married	841	95.4	
Not married	41	4.6	
Monthly household income			
Less than 100 USD	369	41.8	
100 USD	430	48.8	
200 USD	79	9	
300 USD or more	4	0.5	
Perceived social support^a^			
Yes	704	79.8	
No	178	20.2	
Support from husband			
A lot	448	50.8	
Some	344	39	
Little	75	8.5	
Never	15	1.7	
Mother’s age M (SD)	28.25 (6.4)		15–60
Number of children M (SD)	4.61 (2.5)		1–16
Age of the youngest child M (SD)	1 (1.4)		0–10
PHQ-9^b^ total score M (SD)	4 (5.1)		0–21
PBA^c^ total score M (SD)	6 (12.8)		0-95

^a^ Question: “Do you have family, relatives, friends, or others who can give you help and support when needed?”

^b^ Patient Health Questionnaire-9 (Depression)

^c^ Parental Burnout Assessment

### The relation between PBA, depression, social support and sociodemographic factors

The results from the regression analysis (Table 2) indicated an overall explained variance of 23% by the variables in the regression model (Adjusted R^2^ = .231, F (7,867) = 38.557, *p* < .001). Neither number of children nor support from the husband were statistically significant predictors of parental burnout (p < .05). Parental burnout was significantly associated with depression and perceived social support, where higher levels of depression were correlated with higher levels of parental burnout. Low perception of social support significantly predicted higher PBA scores. Further, being unmarried was associated with higher PBA scores, as well as having older children. Lastly, lower monthly household income was associated with higher PBA scores.

**Table 2:** Regression model for PBA^a^ total score.

	Standardised *β*	t	*p*-value
(Constant)		8.862	< .001
Marital status^b^	-.167	-5.523	< .001
Number of children	-.052	-1.671	.095
PHQ9^c^ Total score	.278	7.018	< .001
Age of the youngest child	.090	2.899	.004
Perceived social support	-.287	-9.204	< .001
Monthly Household income	-.074	-2.312	.021
Support from husband^d^	.028	.717	.474

^a^ Parental Burnout Assessment

^b^ Reference category: *not married*

^c^ Patient Health Questionnaire-9 (Depression)

^d^ Scaled high to low

## Discussion

The results of this study demonstrated that the overall level of parental burnout among Somali mothers in Somalia was low. In addition, certain factors were associated with the condition (i.e., marital status, household income, the age of the youngest child, depression, and perceived social support). This study was the first to examine parental burnout among mothers in Somalia, as such it constitutes an important extension of previous research. From previous studies it has been shown that the score of the condition varies [25]. By comparing the findings from this study with previous research, the score of parental burnout among Somali mothers is lower. And much like the results from this study, other Sub-Saharan countries (e.g., Togo and Cameroon), as well as other countries in the global South (e.g., Thailand and Pakistan) presented a lower mean score in comparison to many other countries [19, 25]. This may from one perspective propose that there are factors in these countries that inhibit the experience of parental burnout, such as parenting cultures [19] and societal values [25]. In African countries, such as Somalia, parenting occurs in a collectivistic culture. As a result, the parental responsibility does not merely fall on the parent but is instead shared with relatives and others in their network [25]. Therefore, it can be hypothesised that sharing the parental responsibilities and tasks with others may be an important resource that has contributed to the low parental burnout among this sample. From another perspective, there may be differences in methodological aspects that contribute to the lower mean scores, such as the population size and the chosen target group in this study, along with the questionnaire used.

There have been scientific uncertainties concerning the correlation between sociodemographic factors and parental burnout [13, 15]. Some studies have concluded that being unmarried [14, 26] and having a lower monthly household income [13] are correlated with higher levels of parental burnout. These results dovetail with the findings from this study. Thus, this may indicate that even in a Somali context, these factors remain important in understanding parental burnout. Interestingly, previous studies have concluded that having a child under five years of age were associated with higher levels of parental burnout [11]. However, this study showed that the level of parental burnout among Somali mothers increased with the child’s age. One possible explanation may be found in how the environmental context can affect the parenting experience, as suggested by Bornstein [27]. In the city of Mogadishu, where civil unrest and terrorist attacks are common, there is a greater responsibility and pressure to raise a child in a safe and nurturing environment [20]. Thus, mothers whose youngest child is of school-age may possibly be faced with more pressure and demands in their parental role due to the child having to leave the house. Thus, the findings from this study become an interesting aspect to consider and may provide a new perspective on the condition. In addition, much like previous research [8, 13], this study demonstrated that parental burnout was significantly correlated with depression. Due to this study being the first to examine this correlation in a post-conflict and Sub-Saharan setting, it may therefore widen the understanding of the condition in other settings, along with stressing the importance of examining other psychological factors to the condition. Interestingly, and in contrast to previous studies, the findings from this study indicated that partner parental support did not significantly correlate with parental burnout. This may be due to how the mother’s role as the primary caregiver who is independently responsible for various domestic duties is widely adopted and accepted [5]. Thus, the support from their husband may not be regarded as an important resource for the mothers. Lastly, since this study is the first to examine the correlation between the condition and perceived social support in a Sub-Saharan context and among a wider population, it constitutes an important extension of research on parental burnout. Moreover, the results from this study dovetail with the only other study [28] examining the correlation between the condition and perceived social support. Thus, this highlights the importance of examining this phenomenon further to create a more grounded knowledgebase. Our findings underscore the importance of providing parenting support for parents who are at risk of experiencing parental burnout or have already indicated symptoms of parental burnout. Therefore, the focus should be on implementing preventive interventions and screening parents during antenatal care.

### Limitations and strengths

While the present study has the merit of providing a broader knowledge base, it is not without limitations. From a methodological point, it is important to note that the PBA measure has not been validated in a Somali-speaking sample. However, the possibility of using the PBA measure was tested through a pilot test, which indicated that the measure functioned well and the questions were comprehensive among the pilot participants. Nonetheless, since the measure has not been formally validated, the results from this study must therefore be interpreted with caution. Another possible limitation in this study is related to the social desirability factor and the stigma around mental health in Somalia. Due to the low literacy levels in Somali, reading the questions and the response options became an inevitable and crucial aspect in this study. This may, however, have hindered some mothers from truthfully answering questions regarding mental health in fear of being stigmatised. To the best of our knowledge, however, this is the first study to examine parental burnout in a post-conflict, Sub-Saharan setting and among the most vulnerable group. As such, it provides an important extension of previous research. In addition, even though the sample consisted of only mothers, it still remained both large and heterogenous. Thus, it provided a larger insight in a bigger and diverse population.

As mentioned in the introduction, there is evidence that other mental conditions, such as anxiety, are related to parental burnout. In the present study, no data on anxiety symptoms or other mental conditions were collected. Hence, the association between parental burnout and other mental conditions apart from depression could not be assessed in this context, which can be seen as a limitation. Further, all data were collected during the Covid-19 pandemic. As different individuals may have responded differently to the outbreak and its direct and indirect effects on everyday life, it is possible that the results would be different in a post-pandemic context. Lastly, with the high response rate in this study it provided both higher data quality and accuracy to this study.

## Supporting information

S1 Checklist Inclusivity in global research. (DOCX)
